# Crystallization of the human tetraspanin protein CD9

**DOI:** 10.1107/S2053230X1801840X

**Published:** 2019-04-02

**Authors:** Rie Umeda, Tomohiro Nishizawa, Osamu Nureki

**Affiliations:** a Department of Biophysics and Biochemistry, Graduate School of Science, The University of Tokyo, 7-3-1 Hongo, Bunkyo-ku, Tokyo 113-0033, Japan

**Keywords:** crystallization, LCP, membrane proteins, CD9, tetraspanins

## Abstract

The tetraspanin family of proteins with four membrane-spanning proteins function in a wide range of physiological processes in higher organisms, including cell migration and proliferation, cell fusion, fertilization and virus infection. Although the recently reported structure of CD81 unveiled the basic architecture of this family for the first time, further structural and functional studies are required in order to understand the mechanistic details of the complicated functions of the tetraspanin-family proteins. In this study, attempts were made to crystallize human CD9, a representative member of the tetraspanin family, and it was demonstrated that the truncation of a variable region in the second long extracellular loop significantly improved crystal growth.

## Abstract 2

A method to obtain improved crystals of the human tetraspanin protein CD9 by protein modification is described.

## 1. Introduction   

The tetraspanins are a family of four-transmembrane-helix proteins that are widely conserved in eukaryotes, with 33 identified human members (Charrin *et al.*, 2009 ▸). The tetraspanin proteins share a common architecture consisting of four transmembrane helices (TM1–TM4) and two extracellular loops, with a short extracellular loop (SEL) between TM1 and TM2, and a large extracellular loop (LEL) between TM3 and TM4 (Zimmerman *et al.*, 2016 ▸; Charrin *et al.*, 2014 ▸). Numerous studies have suggested that tetraspanin proteins interact with other functional proteins, such as adhesion proteins, cell growth factor receptors and intracellular scaffold proteins, and organize a ‘tetraspanin-enriched microdomain’ (TEM) where tetraspanins and their ‘partner proteins’ form signaling platforms in cells (Hemler, 2005 ▸; Yáñez-Mó *et al.*, 2009 ▸). Owing to their diverse ‘partner proteins’, tetraspanins are involved in a wide range of cell functions (Hemler, 2003 ▸; Levy & Shoham, 2005 ▸). Notably, since tetraspanins play important roles in cell proliferation and Hepatitis C virus (HCV) infection (Pileri *et al.*, 1998 ▸), they are considered to be drug targets for the treatment of cancer or for protection from HCV infection (Hemler, 2014 ▸). The crystal structure of the tetraspanin protein CD81 has recently been reported (Zimmerman *et al.*, 2016 ▸), which revealed the basic architecture of this protein family, but further structural and functional studies are required in order to elucidate how tetraspanin proteins function in cells.

CD9 is the best-characterized member of the tetraspanins and is expressed in various tissues and cells (Reyes *et al.*, 2018 ▸; Jankovičová *et al.*, 2015 ▸). In particular, CD9 plays an essential role in fertilization. CD9-knockout female mice exhibit an infertility phenotype caused by the failure of sperm–egg fusion (Miyado *et al.*, 2000 ▸; Le Naour *et al.*, 2000 ▸; Kaji *et al.*, 2000 ▸). Despite the physiological importance of CD9, the detailed molecular mechanism by which CD9 regulates cell fusion and other signal transduction pathways has remained unclear. To understand this mechanism, the structure of CD9 and structure-based functional analyses have long been awaited. In this study, we designed various constructs of CD9 by modifying the flexible loop region of the LEL, and revealed that truncation of the LEL facilitates the crystallization of CD9 in lipidic cubic phase. This method could be applied to other tetraspanin proteins to facilitate structural studies of this unique protein family.

## 2. Materials and methods   

### 2.1. Macromolecule production   

The *Homo sapiens* CD9 gene (UniProt accession P21926) was cloned into a modified pFastBac1 expression vector (Invitrogen), which includes an N-terminal His_8_ tag, a GFP tag and a Tobacco etch virus (TEV) protease cleavage site. The DNA fragment encoding CD9 was PCR-amplified using PrimeSTAR Max DNA Polymerase (Takara). The PCR product was inserted into the KpnI and EcoRI sites of the vector. The five extracellular loop residues and the three C-terminal residues of CD9 were deleted by a PCR-based method. For mercury derivatization, a cysteine mutation was introduced by site-directed mutagenesis (Table 1 ▸).

The modified CD9 construct was expressed in Sf9 cells using the Bac-to-Bac baculovirus expression system (Invitrogen). The CD9 expression plasmid was transformed into DH10Bac competent *Escherichia coli* cells (Invitrogen) to generate a recombinant bacmid. The isolated bacmid DNA was transfected into 50 ml of Sf9 cells at a density of approximately 3 × 10^6^ cells ml^−1^ using FuGENE HD (Promega) and the cells were incubated at 27°C for four days. After incubation, the cells and large debris were removed by centrifugation (2600*g*, 5 min) and the clarified supernatant was used for the subsequent protein expression.

For protein expression, Sf9 cells cultured in Sf-900 II medium (Invitrogen) were infected at a density of approximately 3 × 10^6^ cells ml^−1^ and incubated at 27°C for 48 h. The cells were collected by centrifugation (5000*g*, 12 min) and the following procedures were performed at 4°C or on ice. The cells were resuspended in 50 m*M* HEPES pH 7.0, 150 m*M* NaCl with protease inhibitors (1.7 µg ml^−1^ aprotinin, 0.6 µg ml^−1^ leupeptin, 0.5 µg ml^−1^ pepstatin and 1 m*M* phenylmethyl­sulfonyl fluoride). The cells were disrupted by sonication and the cell debris was removed by centrifugation (4000*g*, 10 min). The supernatant was ultracentrifuged (186 000*g*, 1 h) and the membrane fraction was collected and resuspended in 50 m*M* HEPES pH 7.0 containing 150 m*M* NaCl.

The membrane fraction was solubilized in 10 m*M* HEPES pH 7.0, 150 m*M* NaCl, 1.5%(*w*/*v*) *n*-dodecyl-β-d-maltoside (DDM), 0.3%(*w*/*v*) cholesteryl hemisuccinate (CHS) and the solubilized proteins were purified using the three following chromatographic steps. The insoluble material was removed by ultracentrifugation (186 000*g*, 30 min). The supernatant was mixed with TALON metal-affinity resin (Clontech) and incubated for 30 min. After incubation, the resin was washed with seven column volumes of 10 m*M* HEPES pH 7.0, 150 m*M* NaCl, 0.1% DDM, 0.02% CHS, 20 m*M* imidazole pH 7.0. The protein sample was then eluted with three column volumes of 10 m*M* HEPES pH 7.0, 150 m*M* NaCl, 0.1% DDM, 0.02% CHS, 300 m*M* imidazole pH 7.0. The eluted sample was mixed with His-tagged TEV protease (purified in-house) to cleave the His_8_-GFP tag and was dialyzed against 10 m*M* HEPES pH 7.0, 150 m*M* NaCl, 0.1% DDM, 0.02% CHS to remove the imidazole. After overnight dialysis, the sample was mixed with 5 ml Ni–NTA Superflow resin (Qiagen) and incubated for 10 min at 4°C to remove the His_8_-GFP tag and TEV protease. The collected flowthrough fraction was then concentrated using an Amicon Ultra filter (30 kDa molecular-mass cutoff, Millipore) and further purified by gel filtration (Superdex 200 Increase 10/300 GL, GE Healthcare) in 10 m*M* HEPES pH 7.0, 150 m*M* NaCl, 0.05% DDM, 0.01% CHS. The peak fractions were concentrated to approximately 15 mg ml^−1^ using an Amicon Ultra filter (molecular-mass cutoff 50 kDa, Millipore). The truncated and cysteine mutants were purified using the same procedure as described above.

### 2.2. Crystallization   

The purified CD9 samples were reconstituted into lipidic cubic phase (LCP) by mixing them with liquefied monoolein (Sigma) in a 2:3(*w*:*v*) protein:lipid ratio using the twin-syringe mixing method (Caffrey & Cherezov, 2009 ▸). For sandwich-drop crystallization, aliquots of the protein–LCP mixture (50 nl) were dispensed onto 96-well glass plates and overlaid with the precipitant solution (800 nl) using a Griffin LCP robot (Art Robbins Instruments). Initial crystallization conditions were searched for using screening kits including MemMeso (Molecular Dimensions). The initial hits were optimized by changing the concentration of each component (Table 2 ▸).

The crystals were harvested using MicroMounts (MiTeGen) or mesh grid loops (MiTeGen) and were flash-cooled in liquid nitrogen. To prepare mercury-derivative crystals, we co-crystallized CD9 with methyl­mercury chloride. Methylmercury chloride was dissolved in DMSO and added to the protein samples at a final concentration of 2 m*M*. After incubation for 20 min on ice, the samples were crystallized by the LCP method as described above.

### 2.3. Data collection and processing   

All diffraction data sets were collected using the micro-focused X-ray beam at BL32XU at SPring-8 (Hirata *et al.*, 2013 ▸). The microcrystals in the loop were identified by a raster scan and analysis by *SHIKA* (Ueno *et al.*, 2016 ▸). Small-wedge data sets, each consisting of 5–30°, were collected from single crystals. The collected data sets were automatically processed with *KAMO* (Yamashita *et al.*, 2018 ▸). All data sets were collected at a wavelength of 1.000 Å. Anomalous diffraction data sets were collected from the mercury-derivative crystals at a wavelength of 1.000 Å. Each data set was indexed and integrated using *XDS* (Kabsch, 2010 ▸), followed by a hierarchical clustering analysis using the correlation coefficients of the normalized structure-factor amplitudes between data sets. Finally, a group of outlier-rejected data sets were scaled and merged using *XSCALE* (Kabsch, 2010 ▸). The data-processing statistics are summarized in Table 3 ▸. One Hg atom site was identified with *SHELXD* (Sheldrick, 2015 ▸). The initial phases were calculated with *AutoSHARP* (Vonrhein *et al.*, 2007 ▸).

## 3. Results and discussion   

**Figure 1:**
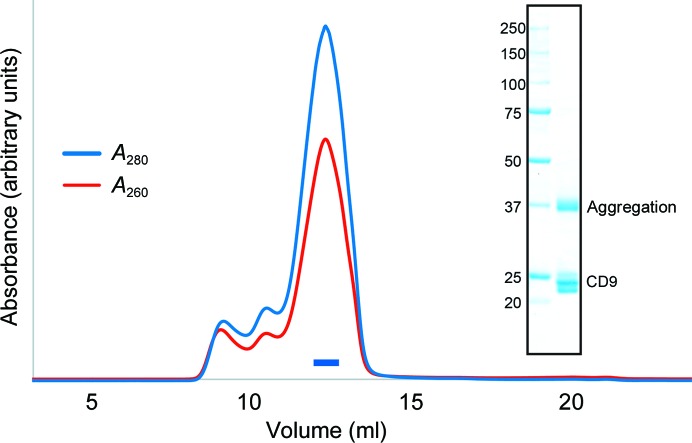
Protein preparation. Size-exclusion chromatogram of wild-type CD9. The blue and red lines indicate the absorbance at 280 and 260 nm, respectively. The blue bar indicates the fractions that were collected and used for crystallization. The inset shows an SDS–PAGE analysis with Coomassie Brilliant Blue staining. Left lane, molecular-mass markers (labeled in kDa); right lane, wild-type CD9. The multiple bands at around 23 kDa represents heterogeneous palmitoylation of the purified CD9 protein, and a higher molecular-weight band at 37 kDa is owing to molecular aggregation during SDS denaturation.

We selected human CD9 as a target for crystallographic analysis and purified the protein by metal-affinity chromatography and size-exclusion chromatography (Fig. 1 ▸). We first crystallized wild-type CD9 (full length, residues 1–228) in lipidic cubic phase (Caffrey & Cherezov, 2009 ▸), and the initial crystals were obtained in a reservoir solution consisting of 36–42% PEG 200, 10–50 m*M* Tris–HCl pH 7.5 or a similar solution containing 10–50 m*M* MOPS pH 6.6 instead of Tris–HCl pH 7.5. Despite repetitive optimizations of the crystallization conditions, these crystals only grew to approximate dimensions of 10 × 10 × 5 µm and diffracted X-rays to a maximum of only 10 Å resolution.

**Figure 2:**
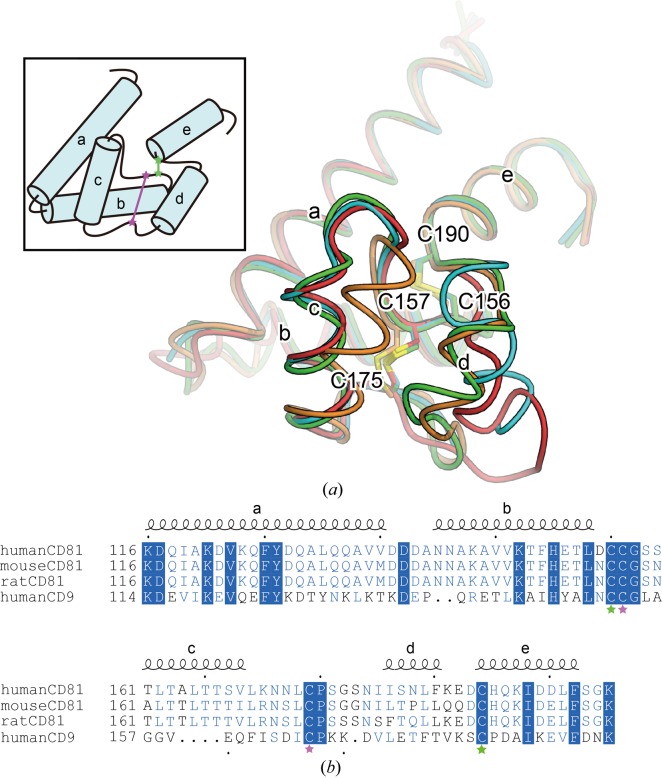
Flexibility of the large extracellular loop. (*a*) Superimposition of the large extracellular loops of the human CD81 structures [PDB entries 1g8q (green and cyan) and 1iv5 (orange and red)], viewed from the extracellular side. The inset shows a schematic diagram, with green and pink stars representing cysteine residues forming intramolecular disulfide bonds. (*b*) Amino-acid sequence alignment of the large extracellular loops of human CD81, mouse CD81, rat CD81 and human CD9. Fully and partially conserved residues are highlighted by blue panels and blue letters, respectively. The secondary structure of CD81 is indicated above the alignment. Residues are numbered according to human CD81. Green and pink stars indicate the cysteine residues shown in the diagram in (*a*).

The sequence of the LEL is rather variable among the tetraspanin subtypes, and thus is considered to mediate their interactions with their partner proteins. Crystal structures of the LEL from CD81, which is closely related to CD9, have been reported (PDB entries 1g8q and 1iv5) and revealed that the LEL contains five short helical segments stabilized by two conserved disulfide bonds (Kitadokoro *et al.*, 2001 ▸, 2002 ▸). Among these structures, the third and fourth helical segments between the second and third cysteine residues adopt rather varied conformations (Fig. 2 ▸), indicating their intrinsic flexibility. Analysis of a sequence alignment between CD9 and CD81 suggested that the LEL of CD9 probably adopts a similar architecture to that of CD81, including this flexible region. Considering the possibility that this flexibility hinders the tight packing interactions within the crystals, we produced truncated mutants of the corresponding LEL region (Leu155–Glu160 or Lys170–Ser180) and performed crystallization trials. Among the tested constructs, only the construct that lacked Thr175–Lys179 (Δ175–179; Table 1 ▸) yielded crystals in LCP, and they diffracted X-rays to 3.5 Å resolution.

**Figure 3:**
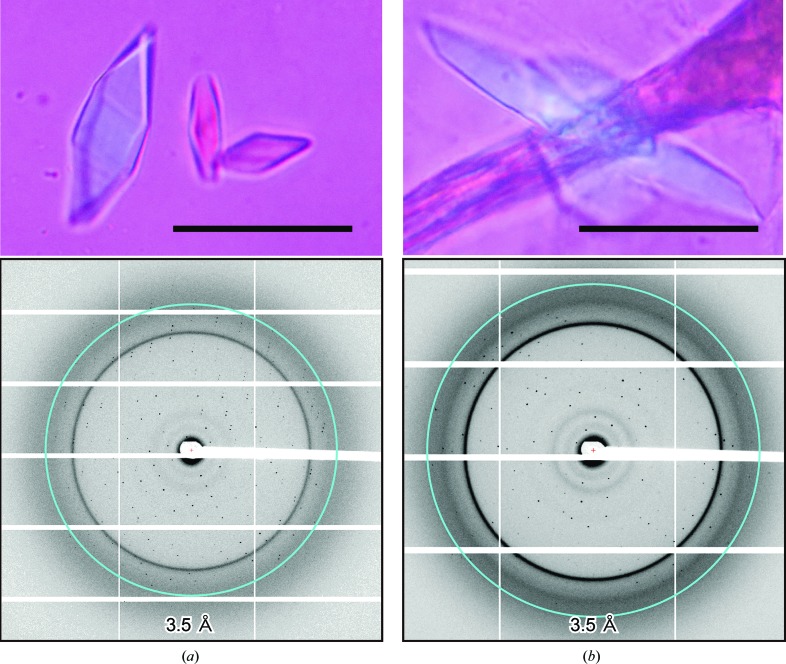
CD9 crystals and X-ray diffraction. Crystals and X-ray diffraction patterns of native CD9_cryst_ (*a*) and mercury-derivatized crystals of CD9_cryst_^I20C^ (*b*). Scale bars represent 30 µm. The rings indicate 3.5 Å resolution.

However, owing to the fragility and low reproducibility of these crystals, we could not collect a complete data set, even though we merged data sets from multiple crystals. To improve the reproducibility of the crystals, we constructed a C-terminally truncated mutant with Δ175–179 and Δ226–228 deletions, which we hereafter refer to as CD9_cryst_. Crystals of CD9_cryst_ were obtained in a reservoir solution consisting of 32–38% PEG 200, 10–50 m*M* MOPS pH 6.5 or a similar solution containing 10–50 m*M* Tris–HCl pH 7.5 instead of MOPS. The crystals grew to maximum dimensions of 50 × 20 × 10 µm, diffracted X-rays to 2.7 Å resolution (Fig. 3 ▸) and belonged to space group *C*222_1_, with unit-cell parameters *a* = 45.18, *b* = 124.83, *c* = 129.23 Å. By merging data sets from multiple crystals, we finally collected a complete data set from the CD9_cryst_ crystals.

We next attempted to prepare mercury-derivatized crystals for experimental phase determination. CD9 contains ten cysteine residues, all of which are predicted to undergo post-translational modifications: four in the large extracellular loop form disulfide bonds and the remaining six at the intracellular ends of the transmembrane helices are heterogeneously palmitoylated (Charrin *et al.*, 2002 ▸). Therefore, to uniformly label the protein we introduced an additional cysteine residue at Ile20, which is predicted to be located at the intracellular end of TM1. This construct, termed CD9_cryst_^I20C^, was co-crystallized with methylmercury chloride, and after optimization crystals of CD9_cryst_^I20C^ grew to maximum dimensions of 50 × 20 × 10 µm under reservoir conditions similar to those used for the native protein. The crystals belonged to the same space group as the native crystals, with similar unit-cell parameters (*C*222_1_, *a* = 45.18, *b* = 125.21, *c* = 129.40 Å; Table 2 ▸), and diffracted X-rays to 3.2 Å resolution (Fig. 3 ▸).

The data-processing statistics are summarized in Table 1 ▸. One Hg-atom site was identified with *SHELXD*, and the initial phases were calculated with *SHARP* by the single isomorphous replacement with anomalous scattering (SIRAS) method, which clearly visualized four transmembrane helices and the extracellular loops. Overall, the current results demonstrate a large improvement in the crystal quality on the modification of the LEL in CD9. While the architecture of the four transmembrane domains is conserved among tetraspanin-family members, the LEL sequences have diverged among the subtypes, suggesting their high flexibility. Notably, the crystallized construct (CD9_cryst_) could rescue the sperm-fusing ability of the eggs from CD9-knockout mice, suggesting that it still retains the critical function of CD9 (manuscript in preparation). Therefore, modification (truncation and/or mutation) of the LEL could be exploited for the crystallization of other tetraspanin proteins, and thus will promote structural studies of the tetraspanin-family proteins.

## Figures and Tables

**Table 1:** Macromolecule-production information

Source organism	*H. sapiens*
DNA source	cDNA Library, Human Mammary Gland (Takara)
Expression vector	Modified pFastBac1
Expression host	Sf9 insect cells
Complete amino-acid sequence of the construct produced†	MPVKGGTKCIKYLLFGFNFI(C)FWLAGIAVLAIGLWLRFDSQTKSIFEQETNNNNSSFYTGVYILIGAGALMMLVGFLGCCGAVQESQCMLGLFFGFLLVIFAIEIAAAIWGYSHKDEVIKEVQEFYKDTYNKLKTKDEPQRETLKAIHYALNCCGLAGGVEQFISDICPKKDVLETFTVKSCPDAIKEVFDNKFHIIGAVGIGIAVVMIFGMIFSMILCCAIRRNREMV

† The truncated residues are underlined. To prepare mercury-derivatized crystals, we introduced a cysteine residue at Ile20, which is shown in parentheses.

**Table 2:** Crystallization

	Native CD9_cryst_	CD9_cryst_^I20C^
Method	Lipidic cubic phase	Lipidic cubic phase
Plate type	96-well plastic sandwich plate	96-well plastic sandwich plate
Temperature (K)	293	293
Protein concentration (mg ml^−1^)	15	15
Composition of reservoir solution	10–50 m*M* MOPS pH 6.5, 32–38% PEG 200 or 10–50 m*M* Tris pH 7.5, 32–38% PEG 200	10–50 m*M* MOPS pH 6.5, 36–38% PEG 200 or 10–100 m*M* Tris pH 7.5, 32–38% PEG 200
Co-crystallized compound (final concentration in mixture with protein solution)	—	2 m*M* methylmercury chloride
Volume of drop (nl)	50	50
Volume of reservoir (nl)	800	800

**Table 3:** Data collection and processing Values in parentheses are for the outer shell.

	Native CD9_cryst_	Mercury-derivatized CD9_cryst_^I20C^
Diffraction source	BL32XU, SPring-8	BL32XU, SPring-8
Wavelength (Å)	1.0	1.0
Temperature (K)	100	100
Detector	EIGER X 9M	EIGER X 9M
Crystal-to-detector distance (mm)	250	300
Rotation range per image (°)	0.1	0.1
Exposure time per image (s)	0.1	0.1
No. of data sets for merging	25	47
Oscillation range per crystal (°)	10–180	10–180
Space group	*C*222_1_	*C*222_1_
*a*, *b*, *c* (Å)	45.18, 124.83, 129.23	45.18, 125.21, 129.40
α, β, γ (°)	90, 90, 90	90, 90, 90
Resolution range (Å)	50–2.70 (2.86–2.70)	50–3.17 (3.43–3.17)
Total No. of reflections	604721	397058
No. of unique reflections	10454	17477
Completeness (%)	100.0 (100.0)	100.0 (100.0)
Multiplicity	57.85	22.72
CC_1/2_	0.998 (0.515)	0.994 (0.740)
〈*I*/σ(*I*)〉	15.79 (1.70)	8.05 (1.70)
*R*_meas_	0.526 (3.167)	0.703 (2.651)
Overall *B* factor from Wilson plot (Å^2^)	48.84	51.34
